# Anti-Aging and Depigmentation Effect of a Hyaluronic Acid Mechanically Stabilized Complex on Human Skin Explants

**DOI:** 10.3390/polym15112438

**Published:** 2023-05-24

**Authors:** Gabriel Siquier-Dameto, Sylvie Boisnic, Pere Boadas-Vaello, Enrique Verdú

**Affiliations:** 1 Dameto Clinics International, 1171 VC Badhoevedorp, The Netherlands; info@dametoclinics.com; 2 Research Group of Clinical Anatomy, Embryology and Neuroscience (NEOMA), Department of Medical Sciences, University of Girona, E-17003 Girona, Catalonia, Spain; pere.boadas@udg.edu; 3 Groupe de Recherche et d’Evaluation en Dermatologie et Cosmétologie (GREDECO), 75116 Paris, France; gredeco@orange.fr

**Keywords:** skin explants, melanin, extracellular matrix, hyaluronic acid, aging, depigmentation

## Abstract

Solar radiation and environmental pollutants are factors that cause changes in the skin that trigger skin aging. The objective of the study is to evaluate the rejuvenating effects of a complex formed by hyaluronic acid supplemented with vitamins, amino acids and oligopeptides in explants of human skin. For this, surplus skin samples have been obtained from donors that have been resected and cultivated on slides with membrane inserts. The complex was administered to some skin explants and the percentage of cells with low, medium and high levels of melanin was evaluated as an indicator of the degree of pigmentation. Other skin segments were irradiated with UVA/UVB, then the product was administered on several slides and the levels of collagen, elastin, sulfated GAG and MMP1 were evaluated. The results show that the administration of the complex significantly reduces the percentage of skin cells with a high melanin content by 16%, and that in skin irradiated with UVA/UVB, there is a decrease in the content of collagen, elastin and sulfate GAGs, and the complex reverses this reduction without changing MMP1 levels. This suggests that the compound has anti-aging and depigmentation effects on the skin, giving a skin rejuvenation appearance.

## 1. Introduction

Hyaluronic acid (HA) is one of the most widely used biopolymers of extracellular matrix components in dermo-aesthetic medicine, with the aim of rejuvenating the skin [1,2,3,4]. In human skin, hyaluronic acid is located in the epidermis, except in the upper granular layer and the stratum corneum, and in the first layers of the dermis (upper and lower dermis) [5,6]. Hyaluronic acid content decreases with aging, both in men’s and women’s skin [7], so its injection improves the aged appearance of the skin, especially to smooth wrinkles. In this context, it has been observed that dermal injection of chemically cross-linked hyaluronic acid (CL-HA) fills spaces, smooths wrinkles and favors collagen formation by skin fibroblasts [8]. CL-HA injections revealed increased collagen formation around the filler [9]. Injection of chemically cross-linked high-molecular-weight HA supplemented with 0.3% lidocaine hydrochloride increases skin volume, improving the aesthetic appearance of the skin [10]. Intradermal injections of cross-linked HA cause a reduction in wrinkles [11]. Injections of non-cross-linked HA also improve skin hydration and elasticity. In the periorbital area, they cause an improvement of between 25% and 50% in skin brightness, texture and turgor [12,13]. The facial application of Viscoderm^®^ Hydrobooster (IBSA SA, Collina d’Oro, Switzerland), slightly cross-linked HA, induces a significant improvement in facial wrinkles and static facial lines [14].

Non-animal stabilized hyaluronic acid (NASHA) injection improves skin texture [15] and skin elasticity [16]. The subcutaneous injection of low-molecular-weight hyaluronic acid fragments mixed with amino acid (HAAM) in aged skin improves the aesthetic appearance of the skin, since it induces the production of collagen III by skin fibroblasts [17]. HA and hydroxyapatite complexes have also been used as skin fillers [18,19,20,21,22]. Combined HA with glycerol improves skin quality by altering viscoelastic skin properties and skin density [23], and HA plus mannitol also is effective for skin hydration [24].

Using CD1 mice, the effect of various HAs has been studied, such as Viscoderm 0.8 a linear HA with a molecular weight of about 1 × 10^6^ Da, Profhilo a hybrid cooperative complex-based compound with low-molecular-weight HA (≈65–110 kDa) and high-molecular-weight HA (≈1.4–2.1 × 10^6^ Da), Profhilo Structura a hybrid cooperative complex-based compound containing low- and high-molecular-weight HA and Aliaxin GP a cross-linked HA (molecular weight 1000 kDa–2000 kDa). This study shows that the degradation kinetics of subcutaneous implants of these four types of HA are 4, 10, 29 and greater than 33 weeks post-injection for Viscoderm, Profhilo, Profhilo Structura and Aliaxin GP, respectively. This suggests that HA fillers with high molecular weight or a mixture of high and low molecular weight have a greater capacity for skin integration [25]. Likewise, in guinea pigs, the intradermal administration of Aliaxin GP, composed of cross-linked HA, and Viscoderm^®^ Skinkò E (SE), composed of non-cross-linked HA supplemented with ions, amino acids and vitamins, shows that the injection of Aliaxin GP has slow reabsorption, inducing scratching of the skin. In the skin of these animals treated with Aliaxin GP, no increase in infiltrates was observed compared to the control group treated with saline solution. In animals treated with Viscoderm^®^ SE, reabsorption is rapid, with slight post-injection erythema being observed. At the histological level, an increase in the deposit of collagen, elastic fibers and proliferation of fibroblasts in the area of administration is observed, with no increase in infiltrates compared to the control group [26].

All these studies are an example of the multiple types of HA used in aesthetic medicine to rejuvenate the skin, ranging from non-cross-linked HA, cross-linked HA, auto-cross-linked HA, as well as low- and high-molecular-weight HA, and supplemented HA with amino acids, ions and vitamins, and preclinical studies provide valuable information on the skin behavior of these HA-based compounds. In summary, this diversity of HA-based products with different molecular weights and rheological characteristics constitutes very useful tools in aesthetic medicine to reduce, delay and partially repair age-related skin changes [27]. In the present study, the effect of treatment with CELLBOOSTER^®^ Glow (Suiselle SA, Yverdon-les-Bains, Switzerland), a revitalizing complex of hyaluronic acid, made up of HA, amino acids (cysteine, glycine, lysine, proline and valine) oligopeptides (glutathione) and vitamins (C, biotin), has been tested. CELLBOOSTER^®^ Glow (CG) is made up of high-molecular-weight hyaluronic acid, not cross-linked and mechanically stabilized by shear deformation and simultaneous pressure. The objective of the study was to demonstrate the efficacy of this revitalizing HA complex in skin depigmentation and anti-aging when injected into the skin of human donors. The results show that in human skin explants, treatment with CG significantly reduces the percentage of cells with a high melanin content, which suggests a depigmentation effect, and that in explants irradiated with UVA/UVB, treatment with CG increases the levels of components of the dermal extracellular matrix, such as collagen type 1, elastin and sulfated GAGs, components that had decreased in irradiated explants. Taken together, these results suggest that CG has a depigmentation and rejuvenation and/or revitalization effect on the skin.

## 2. Materials and Methods

### 2.1. Human Skin Samples Collection and In Vitro Maintenance of Skin Explants

In the present study, skin samples from four female donors between 29 and 57 years of age have been used. The human skin used is excess skin from abdominoplasty (skin #1; 50 years old), from tight lift (skin #2; 57 years old) and breast reduction (skin #3, 30 years old, and skin #4, 29 years old). The donor women signed an informed consent in which they accepted that the resected skin from their interventions could be used for scientific purposes. All procedures performed in this study were in accordance with the ethical standards of the Helsinki Declaration.

In the first hour after excision of the skin, under a laminar flow hood and under sterile conditions, the skin explants were washed with a PBS–antibiotic solution, and the subcutaneous fat and lower dermis were mechanically removed using a surgical scarpel. Under these conditions, the skin was cut into small fragments of approximately 1 cm^2^, which were placed inside plate inserts with a 3 µm pore membrane (# 10769-210; VWR, Rosny-sous-Bois, France) in 12-well culture plates (Costar, VWR, France). The culture medium was placed at the bottom of the wells, ensuring skin survival by slow diffusion between the two compartments through the porous membrane. Medium changes were made 3 times per week. The culture medium used was Dulbecco’s Modified Minimal Essential Medium (DMEM; Life Technologies, Saint-Aubin, France) enriched with antibiotics (penicillin 100 μg/mL, streptomycin 100 μg/mL and amphotericin B 250 μg/mL; Life Technologies), bovine pituitary extract (Life Technologies), L-glutamine (200 μg/mL; Sigma-Aldrich-Merck, Saint-Quentin-Fallavier, France) and fetal calf serum (Sigma-Aldrich-Merck). The culture plates were placed in a humidified incubator in a 5% CO_2_ atmosphere at 37 °C. In each experimental series, the control and test conditions were compared between skin fragments from the same donors [28,29].

### 2.2. Experimental Series and Experimental Design

The effect of CELLBOOSTER^®^ Glow has been tested in two experimental series of human skin explants. In the first experimental series, the depigmentation effect has been analyzed, for which 3 administrations of 10 µL per cm^2^ of the product were injected in the superficial dermis and in the upper part of the middle dermis, of the skin samples. For each donor, duplicates of skins treated with CG and control skins non-treated were made. The day of the injection was considered as day 0 (D0), and the depigmentation effect was evaluated during the following 12 days post-injection. Subsequently, skin samples were formalin-fixed and histologically processed to detect melanin pigment.

In the second experimental series, the anti-aging effect of CG was studied, using a model of aging by ultraviolet radiation. For this, the skin samples were irradiated with ultraviolet A light (UVA; 8 J/cm^2^) and ultraviolet B light (UVB; 1 J/cm^2^) for 60 min by UVA and 2 min by UVB. The source of ultraviolet irradiation was a Vilber Lourmat simulator (Vilber Lourmat, Marnes-la-Vallée, France) fitted out with a UVA irradiation source (365 nm) composed of Vilber Lourmat tubes T-20.L-365 mercury vapor tubes, low pressure. The UVB irradiation source (312 nm) is composed of Vilber Lourmat tubes T-20.L-312 mercury vapor tubes. A radiometer was associated with a microprocessor programmable in energy (J/cm^2^). The skin samples have been irradiated with UVA and UVB because they are the ultraviolet radiations that have the greatest penetrating capacity in the skin, ranging from the epidermis to the dermis, and even the hypodermis. These ultraviolet radiations generate free oxygen radicals, and this oxidative stress is responsible for skin aging [30,31,32]. The day of radiation of the skin samples was considered day 0 (D0). After the ultraviolet radiation session, the skin samples received 3 injections of 10 µL/cm^2^ in the superficial dermis and in the upper part of the mid-dermis of CG. Irradiated non-treated skin and non-irradiated skin also were used as controls. The anti-aging effect of the skin has been studied during the 12 days following irradiation. Between days 1 and 4 (D1-D4), the culture media were collected, mixed and frozen. These media were used for the analysis of metalloproteinase type 1 (MMP1) activity. On day 4 (D4), skin fragments were frozen, which were used for the analysis of glycosaminoglycans (GAGs). Finally, on day 12 (D12), the skin fragments were frozen and used for pro-collagen-I and elastin analysis.

### 2.3. Histological Evaluation of the Melanin Pigment of the Skin

Skin samples treated with CG and control skin samples were fixed in formalin and subsequently embedded in paraffin. The paraffin blocks with the skin inside were cut to a thickness of 4 µm and collected on pregelatinized slides. Histological sections of skin were stained with the method of Fontana-Masson silver (FMS) stain. FMS stain is a histochemical technique that oxidizes melanin and melanin-like pigments as it reduces silver [33]. This histochemical method has been used to determine the degree of depigmentation induced by CG. For this, the histological sections have been deparaffinized and rehydrated with distilled water. Next, the Fontana-Masson stain kit (#HT200-1KT; Sigma-Aldrich-Merck) was used, and the procedure described by the manufacturer was followed. Finally, the histological sections were dehydrated in increasing solutions of ethanol, bathed for 5 min in xylene, and coverslips were mounted with DPX (#06522; Sigma-Aldrich-Merck).

A quantitative numeration of cells containing melanin pigments (black stain) was made under an optical microscope at ×400 magnification on about 300 basal cells of the epidermis. Three types of cells were counted: unpigmented cells or cells presenting rare melanin pigments isolated in the cytoplasm (score 1); cells presenting moderate melanin pigment in all over the cytoplasm (score 2) and cells presenting important melanin pigment in all over the cytoplasm (score 3). Then the epidermal melanin content was calculated as the percentage of the epidermal cells in each cell type counted.

### 2.4. Biochemical Evaluation of Skin Samples

#### 2.4.1. Biochemical Assay of Type 1 Pro-Collagen

During the aging process, the metabolism of fibroblasts is reduced. The objective of an anti-aging product such as CG is to stimulate this metabolism in order to achieve collagen and other extracellular matrix molecules synthesis. Thus, the analysis of the excretion of the single-stranded molecule of type I alpha 1 pro-collagen, before the formation of the triple helix, constitutes an interesting approach to evaluate an anti-aging effect. For it, the skin fragments collected at D12 were weighed and then put in PBS (0.1 M, pH = 7.4) After grinding with a potter, the amount of type I pro-collagen (μg/mL) was evaluated by an ELISA method (# DY6220-05; Human Pro-Collagen I alpha 1, BioTechne), following the procedure described by the manufacturer. The final result was expressed in pg of type 1 pro-collagen/mg of biopsy.

#### 2.4.2. Biochemical Assay of Elastin

Elastin, the main component of elastic fibers, provides stretch and elasticity to the skin. During the aging process, the synthesis of elastin decreases which impacts skin structure, function and youthful appearance. The objective of an anti-aging product such as CELLBOOSTER^®^ Glow is also to stimulate the synthesis of elastin. Therefore, the assessment of the elastin content in skin samples constitutes an interesting contribution to assess the anti-aging effect.

After the 12-day survival period, insoluble elastin was extracted from skin samples with 0.25 M oxalic acid at 100 °C as soluble alpha-elastin polypeptide fragments. After centrifugation to remove undigested tissue, the Fastin Interchim Elastin Assay Kit (#F200; Biocolor, Montluçon Cedex, France) was used, following the manufacturer’s procedure. Finally, the amount of soluble elastin was measured by a spectrocolorimetric assay method at 513 nm. To compare the different results, the quantity of elastin was related to the quantity of total proteins in the sample. The protein concentration (μg/mL) was determined spectrophotometrically at 562 nm, using the Pierce BCA protein assay kit (# 23225; ThermoFisher, Waltham, MA, USA), and following the procedure indicated by the manufacturer. Finally, results were expressed in μg elastin/mg protein.

#### 2.4.3. Biochemical Assay of Sulfated Glycosaminoglycans

In this assay, frozen skin fragments from day 4 (D4) have been used. These fragments were enzymatically digested with papain at 50 °C, overnight. And then, the activity of the fibroblasts to synthesize sulfated GAGs was evaluated by a spectrocolorimetric method at 656 nm, using the Sircoll blyscan GAGs kit (#AA4881; Bicolor, Montluçon Cedex, France), and following the procedure indicated by the manufacturer. To compare the different results, the amount of GAGs was related to the amount of total protein in the sample. The assay of the protein concentration was carried out spectrophotometrically at 562 nm, using the Pierce BCA protein assay kit (# 23225; ThermoFisher, Waltham, MA, USA), and following the procedure indicated by the manufacturer. The results were expressed in μg of sulfated GAGs/mg protein.

#### 2.4.4. Biochemical Assay for the Analysis of Metalloproteinase Type 1 (MMP1) Activity

Metalloproteinases are enzymes involved in the degradation of macromolecules of the extracellular matrix. The interstitial collagenase of fibroblasts MMP1 is specific to type I, II and III fibrillar collagens. MMP1 is first secreted into the culture medium by the fibroblast in its zymogenic form, pro-MMP1, which is then activated into MMP1 by the action of other proteinases.

In this assay, the culture media collected between days 1 and 4 (D1-D4) have been used. This culture medium has been thawed and centrifuged at 900 rpm to remove potential cell debris and other impurities. The amount of MMP1 (pg/mL) contained in the supernatant has then been analyzed by the ELISA technique with a spectrophotometric reader at 450 nm, and using the Human MMP-1 (Matrix Metalloproteinase 1) ELISA Kit (# E-EL-H6073; Elabscience Biotechnology Inc, Houston, TX, USA), and following the procedure described by the manufacturer. The final result was expressed in pg of MMP1/mg of biopsy.

### 2.5. Statistical Analysis

The mean and standard deviation (SD) were calculated in tests performed in doublet in 4 donors in duplicate (8 values). After checking the normality of the groups using the Shapiro–Wilk test, the comparison of the parameter was made with the Student’s T test (alpha risk of 5%). If the groups did not follow a normal distribution, a Wilcoxon test (5% alpha risk) was performed. The IBM SPSS 25.0 statistical package for Windows (IBM Corp. Released 2017; Armonk, NY, USA) was used for the statistical analysis.

## 3. Results

### 3.1. Treatment with CELLBOOSTER^®^ Glow Significantly Reduces the Percentage of Skin Cells with High Melanin Content

**Figure 1:**
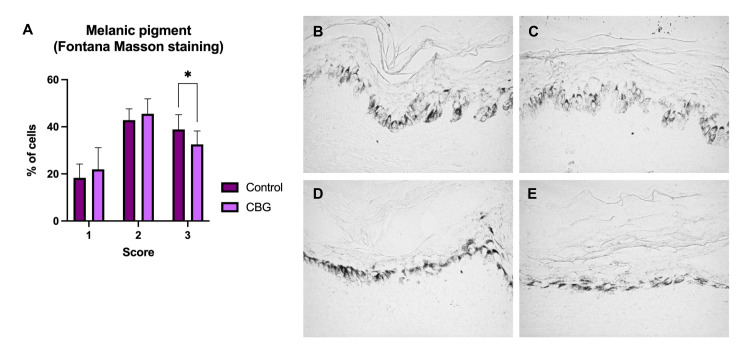
Histological results of skin pigmentation: (**A**) Histogram of the percentage of cells with low (score 1), medium (score 2) and high (score 3) melanin content in both experimental groups (Control, CBG). Values are mean ± standard deviation (*n* = 8 values). * *p* < 0.05 compared to the control group. (**B**,**C**) Light microscope images of skin donor #1, and (**D**,**E**) images of skin donor #2. Images (**B**,**D**) correspond to the control, and images (**C**,**E**) after CG treatment. Comparatively, a slight decrease in black marking (cells with melanin) can be observed in images (**C**,**E**) compared to images (**B**,**D**). All images were captured at ×400 magnification.

In human skin explants, three injections of CG were administered. The percentage of cells with a melanin score of 1 was 18.30% and 21.93% in the control and treated groups, respectively. No significant differences were observed between both groups (*p* > 0.05). Regarding the percentage of pigmented cells with a score of 2, it was 42.83% and 45.52% in the control and treated groups, respectively. Neither were significant differences observed between both experimental groups (*p* > 0.05). Finally, the percentage of cells with a score of 3 was 38.87% and 32.55% in the control and treated groups, respectively. For this score, significant differences were observed between both experimental groups (*p* < 0.05) (Figure 1). CG treatment does not reduce the percentage of skin cells with low and medium melanin content (scores 1 and 2); however, it does significantly reduce the percentage of skin cells with significant melanin content (score 3). This reduction is approximately 16%. Taken together, these results indicate that CG treatment slightly but significantly reduces skin pigmentation. This indication may favor a less-aged cosmetic appearance.

### 3.2. In Skin Treated with Ultraviolet Radiation, Treatment with CELLBOOSTER Glow Significantly Increases the Content of Pro-Collagen Type I, Elastin and Sulphated GAGs, but without Causing Changes in MMP1 Levels

**Figure 2:**
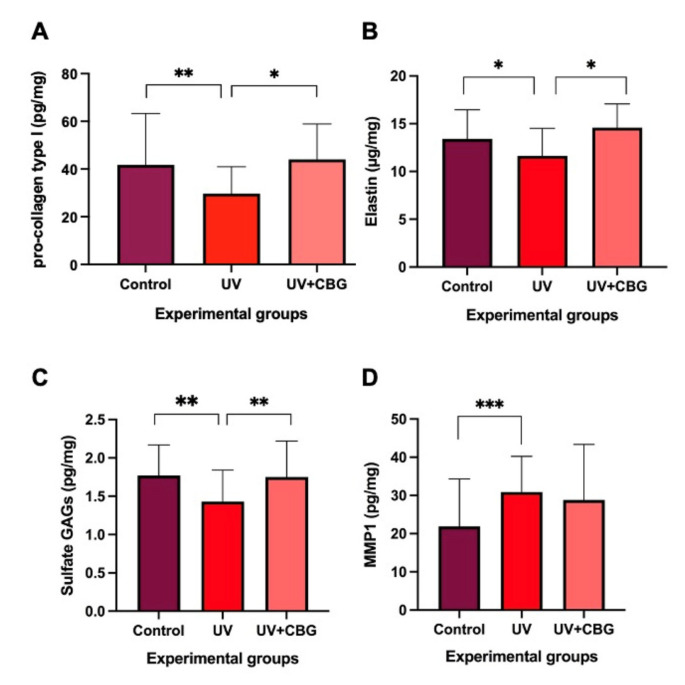
Biochemical results of skin irradiated with UVA/UVB and treated with CELLBOOSTER*^®^* Glow (CG): (**A**) Histogram of the results of the content of pro-collagen type I in control, irradiated with ultraviolet, and in skin irradiated with ultraviolet and treated with CG; (**B**) histogram of the elastin content and (**C**) of sulfated GAGs, in the skin in the same three experimental groups; (**D**) histogram of MMP1 activity and/or content in the skin of the three experimental groups. * *p* < 0.05; ** *p* < 0.01; *** *p* < 0.001. Values are mean ± standard deviation (*n* = 8 values).

Ultraviolet irradiation causes a significant decrease in the levels of type I pro-collagen, elastin and sulfate GAGs in human skin explants compared to the control, which corresponds to non-irradiated skin (Figure 2A–C), while it significantly increases the levels of MMP1 compared to the control (Figure 2D).

When UV-irradiated skin is treated with CG, the levels of pro-collagen type 1, elastin and sulfated GAGs increase compared to irradiated skin without treatment, to levels similar to those observed in the control group (Figure 2A–C). However, MMP1 levels remained similar between the groups of skin irradiated with and without CG treatment (Figure 2D). Ultraviolet irradiation of the skin caused a reduction of 28.9%, 13.2% and 19.2% in the content, respectively, pro-collagen type 1, elastin and GAGs, compared to what was observed in the control group. Likewise, in ultraviolet-irradiated skin, treatment with CG caused a 47.9%, 25.3% and 22.4% increase in the content of pro-collagen type 1, elastin and GAGs, respectively, compared to what was observed in untreated irradiated skin (UV group). In skin irradiated with ultraviolet, a significant increase of 40.9% in the level of MMP1 was observed, compared to what was observed in the control, while in the UV + CBG group, a non-significant reduction of 6.7% was observed compared to the UV group. Taken together, these biochemical results suggest that ultraviolet irradiation of the skin causes changes compatible with aging that manifest as a reduction in the content of pro-collagen type 1, elastin and sulfated GAGs, as well as an increase in MMP1 activity, and the CG treatment reverses these changes by increasing the levels of these extracellular matrix components, except for MMP1 activity, which can be attributed to its rejuvenating effect. In summary, treatment with CG in skin irradiated with ultraviolet radiation increases the levels of pro-collagen type 1, elastin and sulfate GAGs, without modifying the activity of MMP1.

## 4. Discussion

In the present study, it has been observed that in human skin explants, treatment with CG significantly reduces skin pigmentation, especially a significant reduction of 16% of cells with high melanin content has been observed. And in skin treated with ultraviolet A and B radiation, CG treatment significantly increases the content of components of the extracellular matrix of the dermis–epidermis, especially collagen type 1, elastin and sulfated GAGs. All these changes observed in the skin after treatment with CG suggest that this treatment promotes skin rejuvenation.

CELLBOOSTER*^®^* Glow (CG) consists of non-cross-linked and mechanically stabilized HA supplemented with amino acids and vitamins, specifically cysteine, glycine, lysine, proline and valine amino acids, glutathione as an oligopeptide, and as vitamins it contains biotin and vitamin C. The observed effects in the present study may be due to the components of CG. In this context, it is known that ultraviolet radiation causes an increase in the levels of enzymes that degrade the extracellular matrix of the skin, such as collagenases, gelatinases and metalloproteinase type I, which leads to a decrease in collagen content and unstructured resynthesis of this collagen [34]. Ultraviolet radiation also causes a reduction in the release of elastase by skin fibroblasts, so the structural changes that this radiation induces on the elastic fibers of the skin are not easily degraded, which become curly and tortuous, losing skin elasticity and maintaining wrinkles [35]. Ultraviolet radiation triggers the aggregation of elastin. Elastin aggregates are not eliminated by the set of extracellular chaperones present in the skin, because ultraviolet radiation also induces the inactivation of these chaperones. Consequently, these elastin aggregates remain in the skin and this decreases its elasticity [36]. Taken together, all these findings suggest that the skin’s elasticity is compromised by ultraviolet radiation from the sun, giving it a more aged appearance, with the formation of wrinkles. Likewise, ultraviolet radiation also induces a reduction in the expression of hyaluronic synthase enzymes (type 1, 2 and 3), transforming growth factor beta-1, and its type II receptor. All this leads to a lower proliferation of fibroblasts, with a lower synthesis and release of components of the extracellular matrix (e.g., HA and collagen) [37,38,39]. Finally, an in vitro study demonstrated that ultraviolet radiation also causes a reduction in elastin content [40] and a reduction of GAGs in cultured human fibroblasts [41]. In the present study, it has been observed that in human skin explants ultraviolet radiation reduces the content of elastin, type I collagen and sulfated GAGs. In turn, it is known that sulfated GAGs play an important role in establishing interactions with other components of the extracellular matrix, allowing them to retain water, thus maintaining the degree of dermal hydration; retain growth factors and act as co-receptors of these growth factors, responsible for the survival of skin cells; and modulate the inflammatory–immune response of the skin by interacting with cytokines–chemokines [42]. Consequently, the reduction of these sulfated GAGs in skin samples subjected to ultraviolet radiation can compromise all these functions, favoring the appearance of skin signs associated with aging.

As in the present study, other previous studies have also observed an increase in MMP1 levels in UV-irradiated skin samples [43,44] and in cultured human fibroblasts [45]. Matrix metalloproteinase type 1 (MMP1) belongs to the collagenase subgroup and its function is to degrade type I and type III collagen [46]. Type I collagen is synthesized and secreted by dermal fibroblasts and represents 80–90% of skin collagen. Fibroblasts also synthesize and release type III collagen, which represents 15% of the collagen in the skin. Both are of the fibril-forming types. Collagen fibers form extensive and robust networks providing the dermis with strength, firmness and elasticity. A collagen fiber is essentially composed of bundles of smaller fibrils. Collagen fibrils are approximately 10 to 300 nm in diameter and several micrometers in length. A collagen fibril is a bundle of triple-stranded collagen molecules (about 1.5 nm in diameter and approximately 300 nm long). This triple-helix, coiled structure is stereo-dynamically favorable to allow strands to be interwoven together and this incredibly robust structure can persist in tissues for many years. The formation of fibers is dependent on interaction with other ECM components including elastin proteins and GAGs [47]. The increase in MMP1 after ultraviolet irradiation of the skin suggests a degradation of these collagen fibers, a loss of mechanical firmness of the skin and the ability to stretch the skin, as well as a decrease in skin elasticity, and all these cause the appearance of signs of skin aging.

Treatment with CG reverses the observed effects of ultraviolet radiation to levels comparable to control. Specifically, the levels of type I collagen, elastin and sulfated GAGs are restored, although it does not cause a decrease in MMP1. The increased levels of biopolymers in the ECM can be explained by a reactivation of skin fibroblasts by the CG components. In vitro studies show that HA promotes the proliferation of fibroblasts [48,49,50,51,52] that secrete a greater amount of type I collagen [52]. HA binds to the CD44 receptor, expressed by dermal fibroblasts [53], and attenuates MMP1 overexpression [54]. HA can also bind the CD44 receptor on keratinocytes [55] inducing the growth, survival and migration of these epidermis cells [56], which implies an acceleration of the epidermal rejuvenation process. Likewise, the interaction of HA with the CD44 receptor of the keratinocyte also favors the formation and release of lamellar bodies into the extracellular space of the epidermis, which favors the maintenance of the degree of hydration and the rejuvenation of the epidermis [57]. Epidermal melanocytes also express CD44 receptors [58], and sulfate GAGs enhance the melanotic phenotype of these cells through binding to CD44 receptors [59]. In a physiological situation, melanocytes have a layer of hyaluronic acid that surrounds them and that prevents them from synthesizing and secreting cytokines and chemokines. Ultraviolet radiation favors hyaluronidases to degrade HA and melanocytes to secrete these pro-inflammatory factors [60]. It is well known that pro-inflammatory factors, especially cytokines, play a crucial role in the manifestation of decreased skin collagen content, decreased skin thickness and dry and wrinkled skin [61]. Therefore, the application of CG, which provides hyaluronic acid, can potentially restore this HA layer in the melanocytes, preventing these epidermal cells from secreting the inflammatory cytokines that trigger the signs of skin aging. Preclinical evidence indicates that the suppression of the synthesis and release of pro-inflammatory cytokines by melanocytes would be mediated by the binding of HA to CD44 receptors [60]. In addition, in vitro studies show that HA prevents the secretion of inflammatory cytokines by keratinocytes induced by ultraviolet radiation [62] and ionizing radiation [63]. Taken together, all these evidences suggest that the HA biopolymer present in CG has a skin rejuvenation effect through its interaction with CD44 receptors, favoring the proliferation of dermal fibroblasts and the release of extracellular matrix proteins (type I and III collagen, sulfated GAGs, elastin) and preventing the release of inflammatory cytokines by epidermal melanocytes and keratinocytes.

CELLBOOSTER*^®^* Glow also contains biotin and vitamin C. Biotin promotes protein biosynthesis in the liver, intestinal wall and skin [64]. Fibroblasts require biotin for their survival and proliferation [65]. In animals subjected to a diet deficient in biotin, alterations in the composition of fatty acids of the skin are observed, such as accumulation of odd-chain fatty acids and abnormal metabolism of long-chain polyunsaturated fatty acids [66,67]. In these animals, a loss of Langerhans cells in the epidermis is also observed, which compromises the innate immune system of the skin [68]. On the other hand, vitamin C plays different biological functions in the skin, such as promoting collagen synthesis and structural stabilization of collagen fibers; it is a powerful antioxidant that neutralizes and removes oxidant molecules; reduces the synthesis of melanin in melanocytes and facilitates the proliferation and differentiation of keratinocytes and the release of lipids that surround the corneocytes from the stratum corneum of the epidermis, thus facilitating the renewal of the epidermis layers [69]. Vitamin C inhibits the hyaluronidase enzymes responsible for the breakdown of hyaluronic acid in the epidermis [70]. Furthermore, skin pigmentation requires multiple steps, namely, the activation of melanocytes, the synthesis of melanin, the transport of melanosomes to the tips of melanocyte dendrites and the transfer of melanosomes from melanocytes to surrounding keratinocytes. It has been shown that vitamin C inhibits melanin synthesis through downregulation of tyrosinase enzyme activity [71]. Vitamin C also interferes with the transport of melanosomes to the tips of the dendrites of melanocytes, since this vitamin reduces the expression of transport proteins of these melanosomes (e.g., kinesin) [72]. All these findings suggest that these two vitamins present in CG promote the activation of dermal fibroblasts and with it the synthesis of new extracellular matrix proteins; they maintain the proliferation of keratinocytes allowing adequate renewal of the epidermis, as well as the lipid content of the stratum corneum of the epidermis, which contributes both to epidermal renewal and to maintaining the degree of skin hydration; they prevent the degradation of the HA that surrounds the melanocytes, preventing the release of inflammatory cytokines and they decrease the synthesis of melanin and the transport of melanosomes to the dendritic ends of the melanocytes, which has a depigmentation effect. All of these changes can contribute to a more rejuvenated appearance of the skin. Regarding skin pigmentation, in the present study, it has been verified that CG reduces the percentage of epidermal cells with a high melanin content and limits the percentage of cells with a low and intermediate content.

Cysteine, glycine, lysine, proline and valine are the amino acids present in CG. Cysteine is an important amino acid in the synthesis of keratins by the keratinocytes of the epidermis. Lysine is an amino acid that is incorporated into skin proteins and post-translational changes of this residue allow the maturation of these skin proteins. The oxidation of lysine allows cross-linking of the collagen fibers, which gives the stretching strength and insolubility of these protein fibers. Glycine and proline are the two most abundant amino acids in collagen fibers. Proline also undergoes post-translational changes allowing the maturation of collagen fibers. Proline is also an amino acid that promotes skin elasticity [73]. Additionally, glutathione that is present in CG is a tripeptide made up of the amino acid glutamate, cysteine and glycine. It has been shown that glutathione (GSH) not only acts as an antioxidant by scavenging free radicals, but it is also involved in pheomelanin formation and regulating melanogenesis [74]. Oral administration of GSH resulted in the lightening of skin color in humans [75]. Moreover, the oxidized form of glutathione (GSSG) was also found to have anti-melanogenic effects in humans [76,77]. All these evidences suggest that GSH present in CG has anti-aging effects on the skin, sequestering free radicals that cause cell damage that accelerate skin aging, as well as reducing the degree of skin pigmentation by having anti-melanogenic effects, all these contributing to skin rejuvenation. GSH may also contribute to the depigmentation results observed in the present study.

The evidence described above suggests that CG components have skin rejuvenating effects, at least on human skin explants. A good part of the changes observed may be due to the effect of hyaluronic acid on the different cellular elements of skin explants and, to a lesser extent, to vitamins, amino acids and oligopeptides. Recently, newer technologies in transdermal delivery systems had become effective [78] for skin rejuvenation. Nevertheless, clinical studies are necessary to corroborate these effects of CG.

## 5. Conclusions

The objective of this study is to evaluate the rejuvenating effects of a stabilized compound formed by HA supplemented with vitamins, amino acids and oligopeptide in human skin explants. Transformations in the skin, which activate skin aging, can be caused by elements such as the sun’s ultraviolet light and atmospheric contaminants. HA is one of the most widely used biopolymers of extracellular matrix components in dermo-aesthetic medicine, with the aim of rejuvenating the skin. The effect of various HAs has been studied in vivo and in vitro. In the present study, the effect of treatment with CELLBOOSTER*^®^* Glow (CG) has been tested when injected into the skin of human donors. CG is made up of high-molecular-weight hyaluronic acid, non-cross-linked and mechanically stabilized. The results show that in human skin explants, treatment with CG significantly reduces the percentage of cells with a high melanin content, which confirms a depigmentation effect of the formula. In explants irradiated with UVA/UVB, treatment with CG increases the levels of components of the dermal extracellular matrix, such as collagen type 1, elastin and sulfated GAGs, components that had decreased in irradiated explants. Taken together, these outcomes suggest that CG has a depigmentation and rejuvenation and/or revitalization effect on the skin. This indication of a slight reduction of skin pigmentation and an increase of ECM components may favor a less-aged cosmetic appearance.

## Data Availability

All data generated or analyzed during this study are included in this published article.
